# Frank Gower Bergin

**Published:** 1950-01

**Authors:** 


### Obituary

### FRANK GOWER BERGIN

Frank Gower Bergin passed away in his sleep on October 25th, after a prolonged period of restricted activity due to cardiac weakness. His father and brother were at one time Directors of Müller's Orphanage; two brothers were doctors, and a sister was for a while Casualty Sister at the General Hospital. With the late James Taylor, he pioneered radiological services in Bristol, working for many years at the Bristol General Hospital, and later at the Infirmary and the Homoeopathic Hospital as well. In those days there were real dangers to be run, but he was careful, and his health did not suffer seriously. During the first world war he also served in France. He had an extensive private practice in radiology, and his portable apparatus was in great request in the surrounding districts, in spite of smashed tubes and difficulties of transport, which made this work vexatious and expensive. When high power X-ray aparatus and radium became available, he handed over these services to the late Bryan Adams, and confined himself to radiodiagnosis.

He had many interests outside the profession. At one time he was a keen and successful photographer, and always an enthusiastic gardener. He had a lively sense of humour, and a genius for friendship and hospitality. His house in Bristol and his bungalow and Severn-side gardens at Walton Down were constantly thrown open for parties of friends, often fifty or more at a time, students being particularly welcome. He was very active in the service of his church at Alma Road, Clifton. His two sons are both members of the profession, one of them specializing in aviation medicine, and the other in radiodiagnosis.

